# Cervical spinal cord hemisection impacts sigh and the respiratory reset in male rats

**DOI:** 10.14814/phy2.15973

**Published:** 2024-03-11

**Authors:** Matthew J. Fogarty, Wen‐Zhi Zhan, Carlos B. Mantilla, Gary C. Sieck

**Affiliations:** ^1^ Department of Physiology and Biomedical Engineering Mayo Clinic Rochester Minnesota USA; ^2^ Department of Anesthesiology and Perioperative Medicine Mayo Clinic Rochester Minnesota USA

**Keywords:** diaphragm, respiration, spinal cord, transdiaphragmatic pressure, ventilation

## Abstract

Cervical spinal cord injury impacts ventilatory and non‐ventilatory functions of the diaphragm muscle (DIAm) and contributes to clinical morbidity and mortality in the afflicted population. Periodically, integrated brainstem neural circuit activity drives the DIAm to generate a markedly augmented effort or sigh—which plays an important role in preventing atelectasis and thus maintaining lung function. Across species, the general pattern of DIAm efforts during a normal sigh is variable in amplitude and the extent of post‐sigh “apnea” (i.e., the post‐sigh inter‐breath interval). This post‐sigh inter‐breath interval acts as a respiratory reset, following the interruption of regular respiratory rhythm by sigh. We examined the impact of upper cervical (C_2_) spinal cord hemisection (C_2_SH) on the transdiaphragmatic pressure (*P*_di_) generated during sighs and the post‐sigh respiratory reset in rats. Sighs were identified in *P*_di_ traces by their characteristic biphasic pattern. We found that C_2_SH results in a reduction of *P*_di_ during both eupnea and sighs, and a decrease in the immediate post‐sigh breath interval. These results are consistent with partial removal of descending excitatory synaptic inputs to phrenic motor neurons that results from C_2_SH. Following cervical spinal cord injury, a reduction in the amplitude of *P*_di_ during sighs may compromise the maintenance of normal lung function.

## 1 INTRODUCTION

The diaphragm muscle (DIAm) is unique to mammals, and as a pump, it provides the major contribution to the generation of transdiaphragmatic pressure (*P*_di_) for ventilatory (breathing) and non‐ventilatory behaviors, including cough, sneeze, and straining maneuvers (Fogarty & Sieck, 2019; Sieck & Fournier, 1989). During ventilatory behaviors such as quiet breathing (eupnea), breaths in response to hypoxia and/or hypercapnia, or breathing efforts against an occluded airway, the *P*_di_ generated is substantially less than the maximum *P*_di_ generated by bilateral phrenic nerve stimulation (*P*_dimax_) (Fogarty et al., 2023; Khurram et al., 2018; Mantilla et al., 2010; Seven et al., 2018; Sieck, 1991). By contrast, the *P*_di_ generated during non‐ventilatory airway clearance (e.g., cough and sneeze) or straining (e.g., defecation) behaviors are much higher, approaching *P*_dimax_ (Fogarty et al., 2023; Khurram et al., 2018; Mantilla et al., 2010; Seven et al., 2018; Sieck, 1991). Often reported, but less empirically characterized in rats are sighs, also known as augmented breaths, which are biphasic with an increased flow/volume and an extended subsequent inter‐breath interval (Cherniack et al., 1981; Golder et al., 2005; van Lunteren et al., 1983). In cats, the post‐sigh breath is muted in duration and tidal volume (Cherniack et al., 1981; Reynolds Jr. & Hilgeson, 1965), whereas both the post‐sigh inter‐breath interval and tidal volume are increased in mice (Voituron et al., 2010) and unaltered in dogs (van Lunteren et al., 1983). This post‐sigh breath effectively serves as a respiratory reset, allowing for the resumption of normal eupnea post‐sigh (Ramirez, 2014; Ramirez et al., 2022).

It has been suggested that the main function of sighs is to prevent lung atelectasis (Housley et al., 1970; Katagiri et al., 1998; Lieske et al., 2000; Ramirez et al., 2022; Riede et al., 2020; Wuyts et al., 2011). Despite being sensitive to hypoxia, hypercapnia, and lung inflation, sighs still occur when carotid bodies are denervated, in vagotomized conditions and when lungs are transplanted (Severs et al., 2022). Sighs are involved in a variety of emotional states in experimental animals including rats and humans (Ajayi & Mills, 2017; Li et al., 2016; Ramirez, 2014; Ramirez et al., 2022; Riede et al., 2020; Severs et al., 2022; Soltysik & Jelen, 2005; Vlemincx et al., 2022). Sighs are often interchangeably used as a discrete behavior (Burns et al., 2019; Fuller et al., 2008; Seven et al., 2014), a basis for normalization for other breath data (Jensen et al., 2019; Mantilla et al., 2011; Seven et al., 2018; Wen & Lee, 2018), and a “filtered” behavior—normalized to greater than twice eupnea for DIAm EMG (Rana et al., 2017) or 2.5–3 times eupnea for plethysmography (Bell et al., 2009; Bell & Haouzi, 2010; Li et al., 2016; Sheikhbahaei et al., 2017). Many studies have used sighs to simultaneously serve as a combination of all these uses (Bell et al., 2009; Bell & Haouzi, 2010; Hernandez‐Torres et al., 2017; Jimenez‐Ruiz et al., 1985; Mantilla et al., 2011; Rana et al., 2017; Seven et al., 2018), severely curtailing any meaningful interpretation.

There is some contention as to whether the *P*_di_ generated during sighs in rats has a “stereotypical” amplitude (e.g., >twice eupnea) or biphasic pattern (Khurram et al., 2017; Seven et al., 2018) similar to mice (Pareja‐Cajiao et al., 2021), or is variable (e.g., amplitudes <twice eupnea [Khurram et al., 2018]) such as in cats (Reynolds Jr. & Hilgeson, 1965). Additionally, little to no attention has been paid to the post‐sigh inter‐breath interval or respiratory reset in rats. The empirical characterization of sighs in rats would provide a basis for interpreting the neurophysiological underpinnings of DIAm motor unit recruitment, which currently stands as either lower than (Khurram et al., 2017, 2018) or greater than occlusion *P*_di_ (Mantilla et al., 2010). The gamut of respiratory neuromotor *P*_di_ generation in the rat ranges ~10‐fold from between eupnea (~10–15 cm H_2_O) to *P*_dimax_ (~100 cm H_2_O). Where sighs fall within this range is currently unknown, as a winnowing of data by inclusion/exclusion criteria obscures interpretation.

Unilateral upper cervical spinal cord hemisection (C_2_SH) severs ipsilateral descending excitatory inputs to phrenic motor neurons (PhMNs) (Rana et al., 2020; Tai & Goshgarian, 1996) severely blunting PhMN and DIAm activation on the injured side (Miyata et al., 1995). As a result, *P*_di_ and *P*_dimax_ is reduced (Fogarty et al., 2023), with ventilation sustained primarily by compensatory activation of the contralateral DIAm (Fuller et al., 2006; Mantilla, Gransee, et al., 2013; Martinez‐Galvez et al., 2016). Although the effect of C_2_SH on *P*_di_ generated during sighs is unknown, C_2_SH reduces DIAm EMG during sighs (Bezdudnaya et al., 2018), sigh tidal volume (Dougherty et al., 2012; Fuller et al., 2008; Golder et al., 2005), and post‐sigh inter‐breath interval (Golder et al., 2005).

The goal of the present study was to examine the impact of C_2_SH on the *P*_di_ generated during sighs as well as inter‐breath intervals before and immediately after sighs. We also report the proportion of “stereotypical” sighs meeting common inclusion criteria (i.e., *P*_di_ amplitudes and a post‐sigh inter‐breath interval of ≥twice eupnea) under normal pre‐injury, C_2_SH, and sham (laminectomy) conditions. We hypothesize that C_2_SH will reduce *P*_di_ amplitude and the duration of the post‐sigh inter‐breath interval.

## 2 MATERIALS AND METHODS

### 2.1 Experimental animals

A total of 16 adult male Sprague‐Dawley (SD) rats (~325 g) obtained from Envigo (Indianapolis, IN) were used in the study. We have previously used male rats to examine the impact C_2_SH, with a highly repeatable pathological and physiological outcome (Fogarty et al., 2023; Mantilla, Gransee, et al., 2013; Sieck et al., 2021). Animals were acclimated for at least 7 days prior to any procedure and maintained on an alternating 12:12 h light–dark cycle with ad libitum access to fresh water and food. For all procedures, anesthesia was administered via intraperitoneal injections of xylazine (10 mg/kg) and ketamine (80 mg/kg).

### 2.2 Experimental design and timeline

Rats were placed into two experimental groups at random, sham laminectomy, or C_2_SH. These groups were further separated by timepoints into pre‐surgery (PRE‐SHAM and PRE‐C_2_SH) and post‐surgery (POST‐SHAM and POST‐C_2_SH). Anesthetized rats were placed in the prone position, where baseline *P*_di_ was measures once depth of anesthesia was stable (absence of palpebral and deep pain reflexes). *P*_di_ measures for both the sham laminectomy and C_2_SH took ~20 min, with the SHAM or C_2_SH surgeries taking ~20–30 min and the final *P*_di_ recording sessions taking ~20 min. The sham group serves as a time control, as occasional redosing of xylazine (3 mg/kg) and ketamine (25 mg/kg) at ~one third of the initial dose was required (3 in 8 of the C_2_SH and 2 in 8 of the SHAM rats). Thus, all pre‐ and post‐surgery measurements were performed in one experimental session.

### 2.3 *P*_di_ measurements

*P*_di_ was measured as the difference between esophageal and gastric pressures, with two 3.5 French solid‐state pressure catheters (SPR‐524; Millar Instruments, Houston, TX) inserted through the mouth and into the esophagus or stomach (Fogarty et al., 2023; Khurram et al., 2018; Sieck & Fournier, 1989), both before (uninjured) and after C_2_SH or sham laminectomy. The abdomen was bound to approximate isometric conditions. Esophageal and abdominal pressure signals were recorded and digitized (400 Hz) with PowerLab 4/35, with *P*_di_ calculated and visualized in real‐time with LabChart 9 (ADInstruments, Colorado Springs, CO). From the *P*_di_ tracings, *P*_di_ amplitude, respiratory rate (and inter‐breath intervals), and inspiratory duration were determined in a manner identical to previous reports (Fogarty et al., 2022, 2023). All behaviors were recorded with initial O_2_ saturation of >90%, in accordance with past studies (Khurram et al., 2019).

### 2.4 C_2_SH surgery

The surgical methods for C_2_SH have been previously described in detail (Beth Zimmer et al., 2015; Fuller et al., 2006, 2008; Miyata et al., 1995; Streeter et al., 2020), with the lesion functionally validated using DIAm EMG (with electrodes implanted 3 days prior to C_2_SH) during the time of surgery and 3 days post‐surgery, in accordance with previous studies (Fogarty et al., 2023; Mantilla, Gransee, et al., 2013; Sieck et al., 2021). Briefly, following a dorsal laminectomy, the C_2_ spinal cord was cut using a surgical microknife, beginning anterior to the dorsal root entry zone fissure and proceeding ventrally taking care to preserve the dorsal funiculus on the right side, ~7 mm from the midline at the level of the dorsal subdural. The sham group experienced a dorsal laminectomy alone, serving as a time control.

### 2.5 Statistical analyses

The data sets comprise eupnea (with ~130 individual breaths assessed per rat per surgery status [range: 102–154]), sigh (identified manually as a biphasic breath, with a superimposed additional effort causing a point of inflection at the plateauing inspiratory phase [Cherniack et al., 1981; Reynolds Jr. & Hilgeson, 1965]), and post‐sigh eupnea (the breath immediately following a sigh). For sighs and post‐sigh breaths, ~8 breaths were analyzed per rat and only during eupneic breathing (range: 5–12), as sigh incidence and amplitudes may be affected by baseline respiratory drive (Jimenez‐Ruiz et al., 1985; Wen & Lee, 2018). With eight rats, we were powered (*α* = 0.05, *β* = 0.80) to detect a significant difference of >20% in sigh *P*_di_ amplitude and the eupneic inter‐breath intervals following C_2_SH, with mean and variability based on our past study of *P*_di_ in SD rats (Khurram et al., 2017). Prism 9 was used for all statistical analyses (Graphpad, Carlsbad, CA). We performed paired *t*‐tests, two‐ or three‐way ANOVAs with a Geiser‐Greenhouse correction (n.b., we did not assume equal variability between data sets) on all outcome measures. All paired data were done between individual rats pre‐surgery (PRE‐C_2_SH) and post‐C_2_SH (POST‐C_2_SH). Similarly, data were paired within behaviors across sham rats pre‐surgery (PRE‐SHAM) and post laminectomy (POST‐SHAM). When appropriate within group differences were evaluated using Bonferroni post hoc tests. When comparing our empirical data generated within this study to the “stereotypical sigh,” we are referring to twice eupneic amplitudes and twice eupneic post‐sigh inter‐breath intervals (Bell et al., 2009; Bell & Haouzi, 2010; Mantilla et al., 2011; Seven et al., 2018). Statistical significance was set at *p* < 0.05, with *p* values reported to three significant figures in the results. In the interest or reliability, rigor and robustness of our work, the coefficients of variation of each data set, the relative changes reported (when statistically warranted) and the effect size of each PRE‐C_2_SH and POST‐C_2_SH comparison are reported in tabular form (Table 1). All data are reported as the mean ± 95% confidence intervals (CI), unless indicated.

**TABLE 1:** Variance and effect sizes between the PRE‐C_2_SH and POST‐C_2_SH states across rat means.

Parameter	Variance (CV%)	Group differences (%; *p*)	Cohen's *d* (effect size)
Breaths per minute	PRE: 14.7; POST: 10.7	NA; *p* = 0.16	NA
Duty cycle (%)	PRE: 11.8; POST: 13.1	NA; *p* = 0.18	NA
*P*_di_ (cm H_2_O) of rat means within behavior	Eupnea PRE: 23.6; POST: 30.4	−42.4%; *p* = 0.0121	2.04 (large)
	Sigh PRE: 14.1; POST: 33.0	−51.9%; *p* = 0.0001	3.47 (large)
	Post‐sigh PRE: 29.8; POST: 31.9	−40.9%; *p* = 0.0291	1.63 (large)
*P*_di_ (% Eupnea) of rat means within behavior	Sigh PRE: 14.5; POST: 14.2	NA	NA
	Post‐sigh PRE: 16.2; POST: 6.4	NA	NA
Breath duration (s), rat means within behavior	Eupnea PRE: 15.5; POST: 12.9	NA	NA
	Sigh PRE: 22.6; POST: 16.0	NA	NA
	Post‐sigh PRE: 16.2; POST: 19.2	NA	NA
Breath duration (% Eupnea), rat means within behavior	Sigh PRE: 25.3; POST: 13.1	NA	NA
	Post‐sigh PRE: 11.3; POST: 9.7	NA	NA
Inter‐breath interval (s), rat means within behavior	Eupnea PRE: 10.7; POST: 14.0	NA	NA
	Sigh PRE: 27.6; POST: 30.7	NA	NA
	Post‐sigh PRE: 28.5; POST: 22.9	NA	NA
Inter‐breath interval (% Eupnea), rat means within behavior	Sigh PRE: 22.0; POST: 21.9	NA	NA
	Post‐sigh PRE: 25.7; POST: 19.2	NA	NA

## 3 RESULTS

### 3.1 Ventilatory parameters following sham or C_2_SH surgery

The C_2_SH lesion used in the present study is highly consistent both structurally and functionally (Fogarty et al., 2023; Fuller et al., 2006, 2008; Mantilla, Gransee, et al., 2013; Miyata et al., 1995; Rana et al., 2020; Sieck et al., 2021; Warren et al., 2018). C_2_SH severed the right lateral and ventral funiculi resulting in the silencing of eupneic DIAm EMG activity on the right ipsilateral side in anesthetized rats. During eupnea, there was no effect of laminectomy or C_2_SH (*F* = 2.2; *p* = 0.16, two‐way ANOVA) on respiratory rate (PRE‐SHAM: 65.8 ± 14.2 breaths per min; POST‐SHAM: 62.5 ± 8.9 breaths per min; PRE‐C_2_SH: 61.3 ± 7.9 breaths per min; POST‐C_2_SH: 57.6 ± 5.2 breaths per min; *n* = 8 per group). There was also no effect of laminectomy or C_2_SH (*F* = 2.0; *p* = 0.18, two‐way ANOVA) on the DIAm duty cycle during eupnea (PRE‐SHAM: 45.8 ± 5.0%; POST‐SHAM: 47.0 ± 10.7%; PRE‐C_2_SH: 44.5 ± 4.4%; POST‐C_2_SH: 49.8 ± 5.4%; *n* = 8 per group).

### 3.2 *P*_di_ amplitude decreases following C_2_SH during eupnea, sigh, and the immediate post‐sigh breath

**FIGURE 1:**
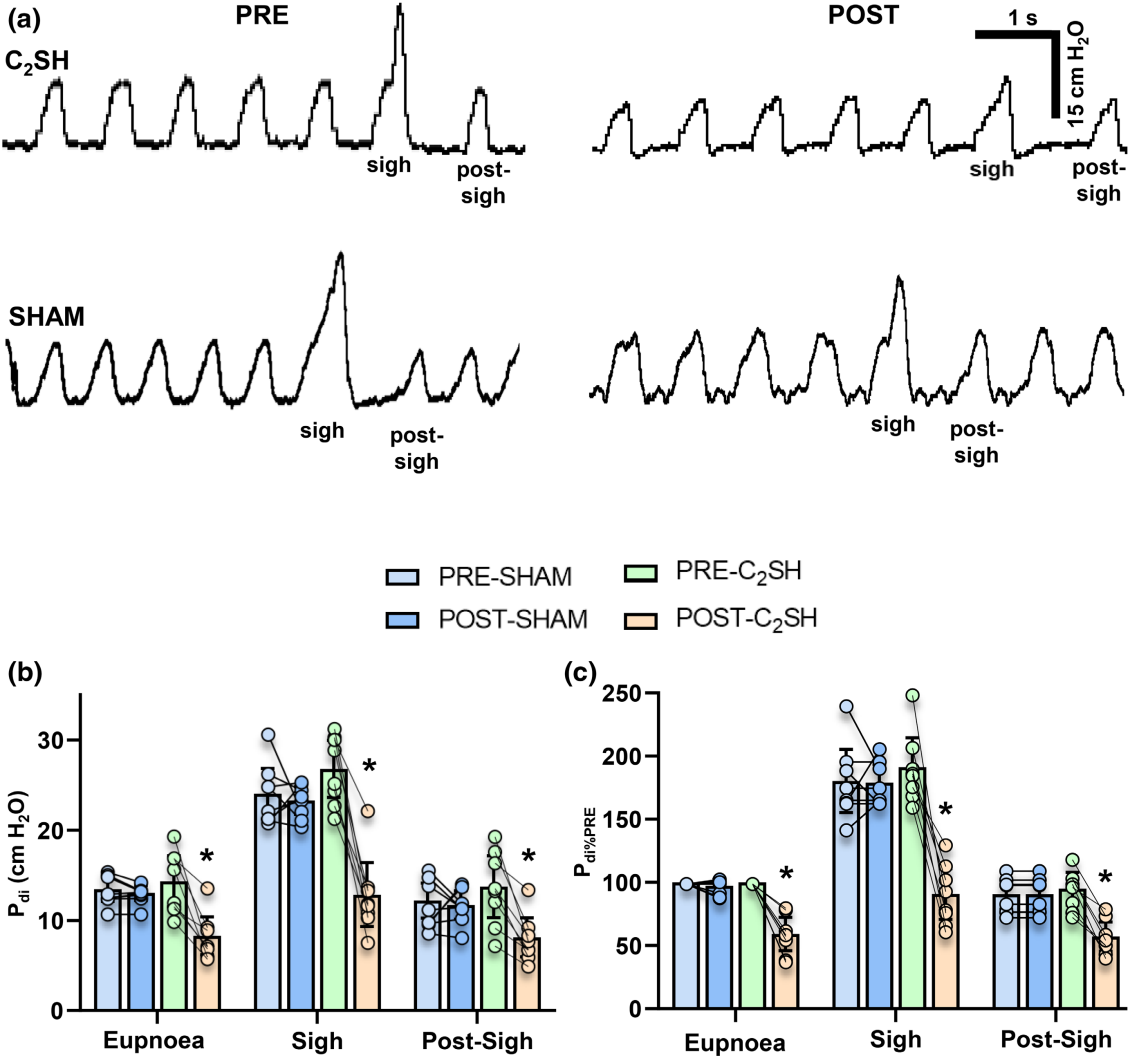
(a) Representative tracing of *P*_di_ during eupnea, sigh, and the immediate post‐sigh breath in PRE‐SHAM, POST‐SHAM, PRE‐C_2_SH, and POST‐C_2_SH conditions. (b) Scatterplot of *P*_di_ (cm H_2_O) showing reduction in the POST‐C_2_SH (orange) compared to the PRE‐C_2_SH (green) condition during eupnea, sigh, and the post‐sigh breath, with no changes between PRE‐SHAM and POST‐SHAM with sigh (except during POST‐C_2_SH) having greater *P*_di_ than all other groups (behavior: *p* < 0.0001; type of surgery: *p* = 0.0002; and time: *p* < 0.0001). (c) Scatterplot of normalized *P*_di_ (*P*_di%PRE_) shows reductions in the POST‐C_2_SH compared to PRE‐C_2_SH condition during eupnea, sigh, and the post‐sigh breath, with no changes between PRE‐SHAM and POST‐SHAM (behavior: *p* < 0.0001; type of surgery: *p* = 0.0002; and time: *p* < 0.0001). All tests are paired three‐way ANOVAs with Bonferroni post‐tests, *n* = 8 for all groups. Each scatter point represents the mean value of each rat, * denotes significant differences within behavior between PRE‐C_2_SH and POST‐C_2_SH groups for *P*_di_ during eupnea (*p* = 0.012), sigh (*p* < 0.0001) and the post‐sigh breath (*p* = 0.029) and for normalized *P*_di%PRE_ during eupnea (*p* = 0.011), sigh (*p* = 0.0001), and the post‐sigh breath (*p* = 0.010).

Amplitudes of *P*_di_ during eupnea, sigh, and the post‐sigh breath (i.e., DIAm motor behaviors) were assessed at PRE and POST timepoints in SHAM and C_2_SH rats (Figure 1a). When the mean of each behavior was calculated for each rat, *P*_di_ amplitude was affected by behavior (*F* = 353.5; *p* < 0.0001), type of surgery (SHAM vs. C_2_SH; *F* = 6.3; *p* = 0.0228), and time (PRE‐ vs. POST; *F* = 24.0; *p* = 0.0002; *n* = 8 per group, three‐way ANOVA; Figure 1b). *P*_di_ amplitude during eupnea was reduced by ~42% in POST‐C_2_SH (8.3 ± 2.1 cm H_2_O) compared to PRE‐C_2_SH (14.4 ± 2.9 cm H_2_O; *p* = 0.0121), with no difference observed between PRE‐SHAM (13.5 ± 1.4 cm H_2_O) and POST‐SHAM (13.0 ± 0.8 cm H_2_O; *p* > 0.99; Bonferroni post‐tests; Figure 1b). *P*_di_ amplitude during sigh was reduced by ~52% in POST (12.9 ± 3.6 cm H_2_O) compared to PRE (26.8 ± 3.2 cm H_2_O; *p* < 0.0001), with no difference observed between PRE‐SHAM (24.1 ± 2.8 cm H_2_O) and POST‐SHAM (23.3 ± 1.5 cm H_2_O; *p* > 0.99; Bonferroni post‐tests; Figure 1b) timepoints. *P*_di_ amplitude during the post‐sigh breath was reduced by ~41% in POST (8.1 ± 2.2 cm H_2_O) compared to PRE (13.7 ± 3.4 cm H_2_O; *p* = 0.0291), with no difference observed between PRE‐SHAM (12.2 ± 1.0 cm H_2_O) and POST‐SHAM (11.7 ± 1.6 cm H_2_O; *p* > 0.99; Bonferroni post‐tests; Figure 1b) timepoints.

Within each rat, when *P*_di_ amplitude during each motor behavior was expressed as a % of PRE eupnea (*P*_di%PRE_), *P*_di%PRE_ was dependent on behavior (*F* = 230.5; *p* < 0.0001), type of surgery (SHAM vs. C_2_SH; *F* = 34.7; *p* = 0.0002), and time (PRE‐ vs. POST; *F* = 55.9; *p* < 0.0001; *n* = 8 per group, three‐way ANOVA; Figure 1c), with *P*_di%PRE_ reduced POST‐C_2_SH compare to PRE‐C_2_SH during eupnea (PRE‐C_2_SH: 100 ± 0%; POST‐C_2_SH: 59 ± 13%; *p* = 0.0108), sigh (PRE‐C_2_SH: 191 ± 24%; POST‐C_2_SH: 91 ± 20%; *p* = 0.0001), and the post‐sigh breath (PRE‐C_2_SH: 95 ± 13%; POST‐C_2_SH: 57 ± 12%; *p* = 0.0096; Bonferroni post‐tests; Figure 1c). There were no differences between PRE‐SHAM and POST‐SHAM in *P*_di%PRE_ during eupnea (*p* > 0.99), sigh (*p* > 0.99), and post‐sigh (*p* > 0.99; Bonferroni post‐tests; Figure 1c).

Note that the amplitude of *P*_di_ for PRE‐C_2_SH sighs was ~double the amplitude of *P*_di_ for PRE‐C_2_SH eupnea, whereas POST‐C_2_SH sigh *P*_di_ amplitude was no different compared to PRE‐C_2_SH eupnea *P*_di_ amplitude (*p* > 0.99; Figure 1c). Overall, these results show the *P*_di_ amplitudes of eupnea, sigh, and the immediate post‐sigh breath were reduced in POST‐C_2_SH rats compared to the PRE‐C_2_SH condition, with no differences in SHAM time controls.

### 3.3 Breath durations are unaltered following C_2_SH during eupnea, sigh, and the immediate post‐sigh breath

**FIGURE 2:**
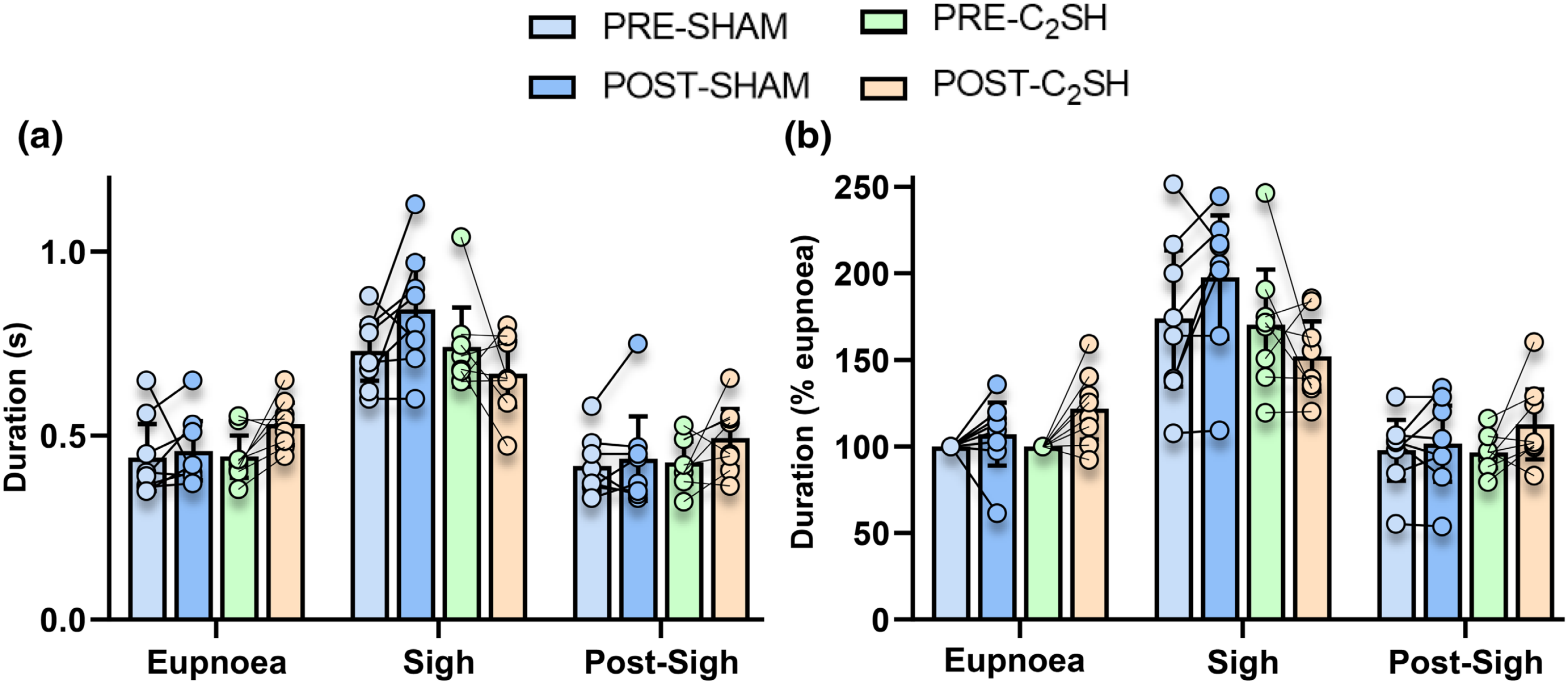
(a) Scatterplot of breath duration (s) showing increased breath duration of sigh compared to eupnea and the immediate post‐sigh breath, regardless of injury (behavior: *p* < 0.0001; type of surgery: *p* = 0.787; and time: *p* = 0.227). (b) Scatterplot of breath durations normalized within rats to PRE‐injury eupnea values shows extended breath durations during sigh compared to eupnea and the post‐sigh breath, regardless of injury (behavior: *p* < 0.0001; type of surgery: *p* = 0.469; and time: *p* = 0.270). All tests are paired three‐way ANOVAs with Bonferroni post‐tests, *n* = 8 for all groups. Each scatter point represents the mean value of each rat.

When the mean of each behavior was calculated for each rat, breath duration was dependent on behavior (*F* = 145.1; *p* < 0.0001), but not type of surgery (SHAM vs. C_2_SH; *F* = 0.02; *p* = 0.787) or time (PRE‐ vs. POST; *F* = 1.5; *p* = 0.227; *n* = 8 per group, three‐way ANOVA; Figure 2a). In all rats, sigh duration was longer compared to eupnea and the post‐sigh breath, regardless of pre‐ or post‐injury timepoint (*p* < 0.05; Bonferroni post‐tests). However, there was no difference between C_2_SH and SHAM rats (Figure 2a).

When breath durations were expressed as a % of PRE eupnea, breath duration was dependent on behavior (*F* = 104.4; *p* < 0.0001) but not type of surgery (SHAM vs. C_2_SH; *F* = 0.3; *p* = 0.469) or time (PRE‐ vs. POST; *F* = 1.3; *p* = 0.270; *n* = 8 per group, three‐way ANOVA; Figure 2b), with sigh having increased durations compared to eupnea and the post‐sigh breath in all rats (*p* < 0.05, Bonferroni post‐tests; Figure 2b). Overall, these results show the breath duration of eupnea, sigh, and the immediate post‐sigh breath was unchanged with C_2_SH or sham.

### 3.4 Inter‐breath intervals are unaltered following C_2_SH during eupnea, sigh, and the immediate post‐sigh breath

**FIGURE 3:**
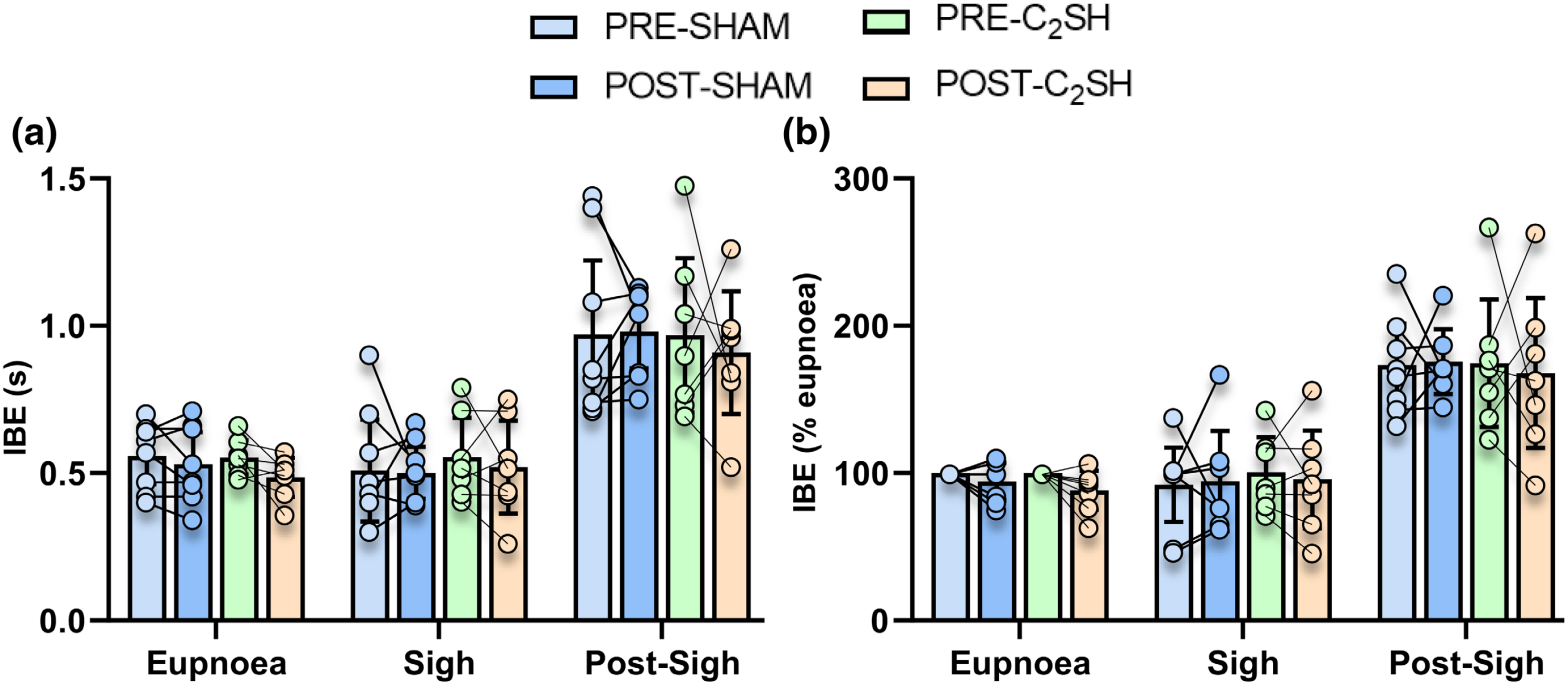
(a) Scatterplot of inter‐breath intervals (s) showing increased inter‐breath intervals (IBE) of the post‐sigh breath compared to all other behaviors, regardless of injury (behavior: *p* < 0.0001; type of surgery: *p* = 0.659; and time: *p* = 0.610). (b) Scatterplot of IBE normalized within rats to PRE eupnea values shows longer inter‐breath intervals in the post‐sigh breath regardless of injury (behavior: *p* < 0.0001; type of surgery: *p* = 0.924; and time: *p* = 0.661). All tests are paired three‐way ANOVAs, *n* = 8 for all groups. Each scatter point represents the mean value of each rat.

When the mean of each behavior was calculated for each rat, the inter‐breath interval was dependent on behavior (*F* = 88.9; *p* < 0.0001), but not type of surgery (SHAM vs. C_2_SH; *F* = 0.2; *p* = 0.659) or time (PRE‐ vs. POST; *F* = 0.3; *p* = 0.610; *n* = 8 per group, three‐way ANOVA; Figure 3a). Inter‐breath intervals of the post‐sigh breath were greater than compared to sigh across all groups (Figure 3a).

When the inter‐breath interval was expressed as a % of PRE eupnea, inter‐breath interval was dependent on behavior (*F* = 89.2; *p* < 0.0001) but not type of surgery (SHAM vs. C_2_SH; *F* = 0.002; *p* = 0.924) or time (PRE‐ vs. POST; *F* = 0.2; *p* = 0.661; *n* = 8 per group, three‐way ANOVA; Figure 3b), with greater post‐sigh inter‐breath interval compared to sigh in all groups (*p* < 0.05, Bonferroni post‐tests; Figure 3b). Overall, these results show the inter‐breath intervals of eupnea, sigh, and the immediate post‐sigh breath were unchanged with C_2_SH or sham.

### 3.5 Sigh variability and comparisons to stereotypical sigh in C_2_SH rats

**FIGURE 4:**
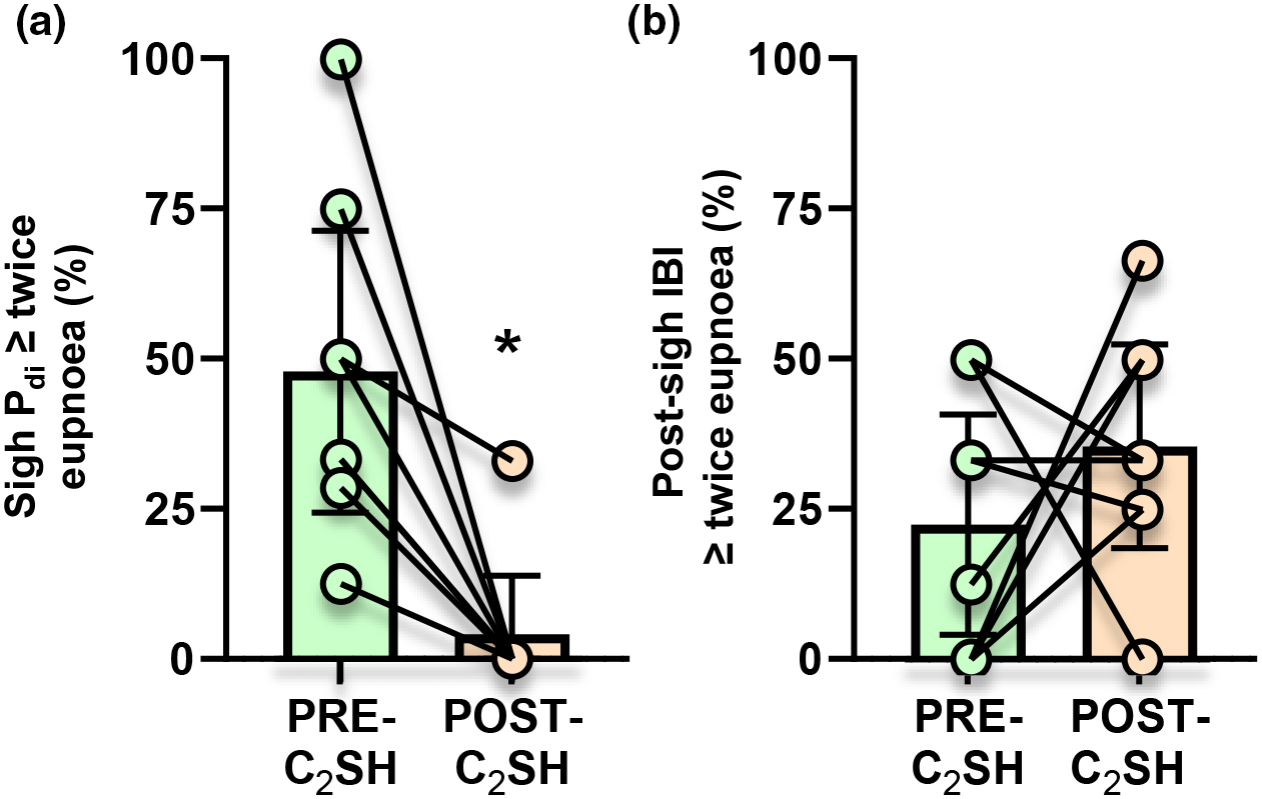
(a) Scatterplot showing the % of all sighs exhibiting “stereotypical” twice eupnea *P*_di_ shows reduction in the % of sighs with canonical *P*_di_ amplitudes in POST‐C_2_SH compared to PRE‐C_2_SH rats (*p* = 0.0078, Wilcoxon matched‐pairs signed‐rank test). (b) Scatterplot showing the % of all sighs exhibiting “stereotypical” twice eupnea inter‐breath intervals (i.e., post‐sigh respiratory reset) of the post‐sigh breath shows no change with injury in the % of sighs with canonical post‐sigh respiratory reset in PRE‐ and POST‐C_2_SH groups (*p* = 0.373, paired *t*‐test). Each scatter point represents the mean value of each rat, *n* = 8 for all groups, * denotes significant differences (*p* = 0.0078) within behavior between PRE‐ and POST‐C_2_SH groups.

As we did not observe a significant effect of time (PRE vs. POST) alone on *P*_di_ from SHAM rats, we ignored these for the paired analyses of PRE‐ and POST‐C_2_SH rats. Variability across rats was readily apparent in both the PRE‐C_2_SH and POST‐C_2_SH injury timepoints (Table 1). Within rats, we characterized the % of all sighs within a rat that had *P*_di_ magnitudes of ≥twice eupnea (i.e., the “stereotypical” sigh amplitude) at PRE‐ and POST‐C_2_SH timepoints (Figure 4a). At the PRE‐C_2_SH timepoint, the mean % of sighs within a rat that exhibited stereotypical *P*_di_ amplitudes was ~48%, ~10 times the % of stereotypical amplitudes observed in the POST‐C_2_SH timepoint (~4%; *p* = 0.0078, Wilcoxon matched‐pairs signed‐rank test; Figure 4a).

Within rats, we characterized the % of all sighs within a rat that had post‐sigh inter‐breath intervals of ≥twice eupnea (i.e., the “stereotypical” apneic period) at PRE‐ and POST‐C_2_SH timepoints (Figure 4b). At the PRE‐C_2_SH timepoint, the mean % of sighs within a rat that exhibited stereotypical post‐sigh inter‐breath intervals was ~22%, with no significant difference observed in the POST‐C_2_SH timepoint (~35%; *p* = 0.373, paired *t*‐test; Figure 4b).

**FIGURE 5:**
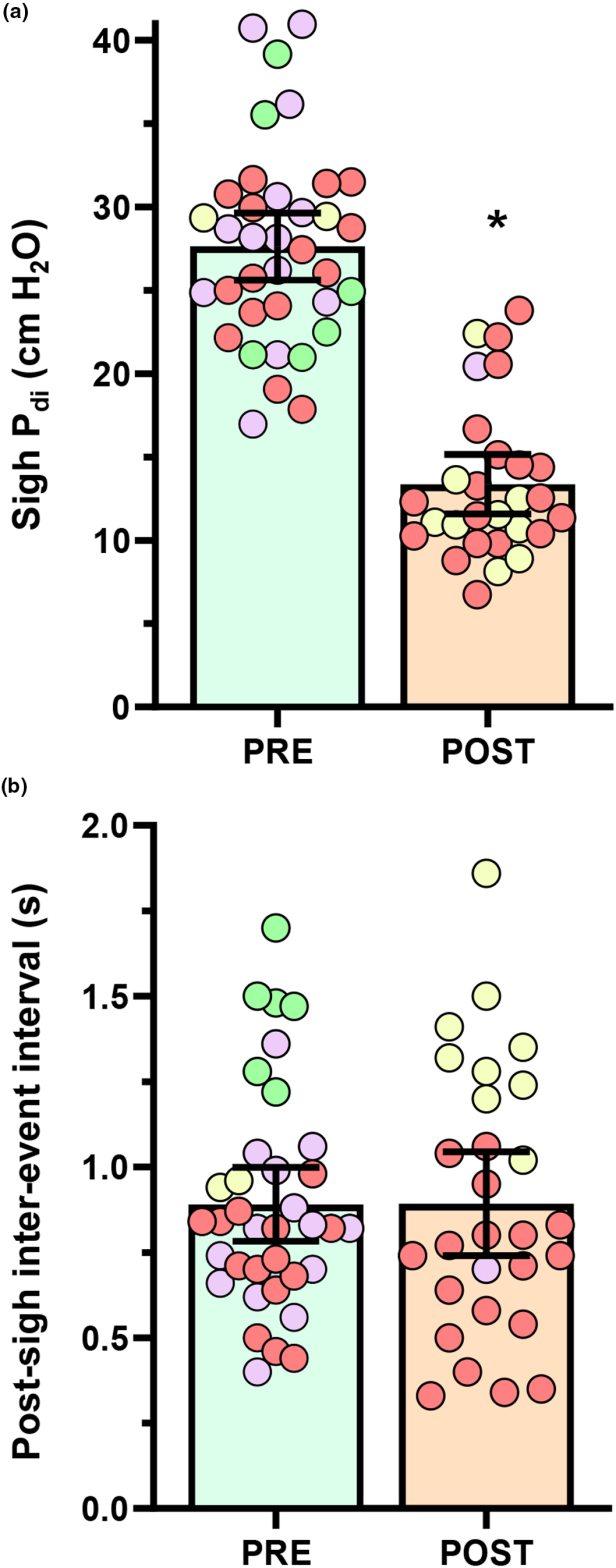
(a) Scatterplot of all individual sigh *P*_di_ (cm H_2_O), showing reduced amplitudes in the POST‐C_2_SH compared to PRE‐C_2_SH timepoints (*p* < 0.0001). (b) Scatterplot of all individual inter‐breath intervals (s) between sigh and the post‐sigh breath, showing no difference between PRE‐ and POST‐C_2_SH timepoints (*p* = 0.979). In all plots, green symbols represent “stereotypical” sighs of >twice eupneic *P*_di_ and post‐sigh respiratory reset, purple symbols represent sighs satisfying “stereotypical” *P*_di_ criteria, yellow symbols represent sighs satisfying inter‐breath interval criteria and red symbols represent sighs where neither criterion was satisfied. All tests are unpaired *t*‐tests, * denotes *p <* 0.0001, *n* = 36 (PRE‐C_2_SH) and *n* = 24 (POST‐C_2_SH).

When all sighs were pooled and considered independent events, the 51% reduction in *P*_di_ amplitude in the POST‐C_2_SH (13.4 ± 1.8 cm H_2_O, *n* = 28) timepoint was apparent compared to PRE‐C_2_SH amplitudes (27.6 ± 2.0 cm H_2_O, *n* = 36; *p* < 0.0001, unpaired *t*‐test; Figure 5a). When all sighs were considered independent events, there was no difference in the post‐sigh inter‐breath interval between PRE‐C_2_SH (0.891 ± 0.110 s, *n* = 36) and POST‐C_2_SH (0.893 ± 0.152 s, *n* = 28) timepoints (*p* = 0.979, unpaired *t*‐test; Figure 5b).

The mean duration, inter‐breath interval, and amplitudes of the sigh and post‐sigh breath were compared to the “stereotypical” sigh at PRE‐ and POST‐C_2_SH timepoints (Figure 5; Table 2), with the reduction in POST‐C_2_SH sigh *P*_di_ amplitude readily observed. Even in the PRE‐C_2_SH condition, there was a marked departure of the sigh and post‐sigh breath from the “stereotypical” sigh. For sigh amplitudes, only 53% (19/36) from the PRE‐C_2_SH and 4% (1/28) from the POST‐C_2_SH timepoints satisfied “stereotypical” criteria, with significant differences between PRE‐ and POST‐C_2_SH timepoints (*p* < 0.0001, Fischer's exact test; Figure 5; Table 2). Likewise, for post‐sigh inter‐breath intervals, only 22% (8/36) from the PRE‐C_2_SH and 32% (9/28) from the POST‐C_2_SH timepoints satisfied “stereotypical” criteria, with no differences between timepoints (*p* = 0.406, Fischer's exact test; Figure 5; Table 2). Incredibly, only 17% of sighs from the PRE‐C_2_SH timepoint and not a single sigh from the POST‐C_2_SH timepoint satisfied both criteria, with significant differences between PRE‐ and POST‐C_2_SH timepoints (*p* = 0.031, Fischer's exact test; Figure 5; Table 2). A rather large portion of sighs satisfied neither criterion 25% at PRE‐C_2_SH (9/36) and 36% at POST‐C_2_SH (10/28) timepoints, with a significant increase between PRE‐ and POST‐C_2_SH timepoints (*p* = 0.001, Fischer's exact test; Figure 5; Table 2).

**TABLE 2:** Number and percentages of sighs exhibiting stereotypical criteria in PRE‐C_2_SH and POST‐C_2_SH conditions.

Group	*P*_di_ ≥ twice eupnea	Post‐sigh inter‐breath interval ≥ twice eupnea	Both criteria satisfied	Neither criteria satisfied	Total sighs
PRE	19 (53%)	8 (22%)	6 (17%)	9 (25%)	36
POST	*1 (4%)*	9 (32%)	*0 (0%)*	*10 (36%)*	28

*Note*: Italicized POST values are significantly different from PRE, Fischer's exact test, *p* < 0.05.

## 4 DISCUSSION

This study was the first to characterize the *P*_di_ generated during sighs and the respiratory reset PRE‐ and POST‐C_2_SH. We present four major findings: (i) *P*_di_ amplitudes, but not breath durations or inter‐breath intervals were altered POST‐C_2_SH during eupnea, sigh, and the immediate post‐sigh breath; (ii) the amplitudes of *P*_di_ during sighs were highly variable, with only ~53% of sigh *P*_di_ amplitudes two times greater than *P*_di_ during eupnea PRE‐C_2_SH, while only ~4% of POST‐C_2_SH sighs exhibited *P*_di_ amplitudes that were > twice eupnea; (iii) while post‐sigh inter‐breath intervals were longer compared to eupnea, only ~22% of sighs PRE‐C_2_SH and ~32% of sighs POST‐C_2_SH displayed inter‐breath intervals that were twice eupnea; and (iv) overall, PRE‐C_2_SH only 17% of all sighs satisfied both “stereotypical” criteria (i.e., amplitudes and inter‐breath intervals greater than twice eupnea), while POST‐C_2_SH “stereotypical” sighs were absent. These findings have important consequences for the study of respiratory function more generally, and for the effects of C_2_SH and DIAm impairments more specifically.

Sighs have been reported in a myriad of reduced and intact experimental preparations and in a variety of species such as humans (Vlemincx et al., 2010; Wuyts et al., 2011), cats (Cherniack et al., 1981; Orem & Trotter, 1993; van Lunteren et al., 1988), dogs (Katagiri et al., 1998; van Lunteren et al., 1983), rats (Ajayi & Mills, 2017; Bell et al., 2009, 2011; Bell & Haouzi, 2010; Fogarty et al., 2023; Fuller et al., 2006, 2008; Golder et al., 2005; Janssen et al., 2000; Jimenez‐Ruiz et al., 1985; Khurram et al., 2017, 2018; Seven et al., 2018), and mice (Li et al., 2016; Pareja‐Cajiao et al., 2021; Voituron et al., 2010). To date, the focus has been primarily on the pulmonary and EMG characteristics of sigh and the post‐sigh breath, with information on rat *P*_di_ during sigh influenced by the “filtering” effect of inclusion criteria. There is also a total absence of quantitative *P*_di_ information on the post‐sigh breath and whether *P*_di_ characteristics of sighs are in accordance with plethysmographic results, where duration and tidal volumes are reduced compared to eupnea in cats (Cherniack et al., 1981; Reynolds Jr. & Hilgeson, 1965), but not dogs (van Lunteren et al., 1983). Previously, sigh *P*_di_ in anesthetized rats was reported to be either less than that generated during airway occlusion in rats (Gill et al., 2015; Khurram et al., 2017, 2018; Seven et al., 2018) or greater than occlusion *P*_di_ (Mantilla et al., 2010). Sigh *P*_di_ in anesthetized cats was also reported to be less than occlusion *P*_di_ (Reynolds Jr. & Hilgeson, 1965). In the present study we evaluated augmented breaths with characteristic waveforms without iexclusion criteria of amplitudes or the duration of the post‐sigh respiratory reset. We found the mean sigh *P*_di_ to be ~27 cm H_2_O compared to 14.4 cm H_2_O during eupnea. The mean sigh *P*_di_ amplitude observed in the present study was moderately higher than that previously reported in Sprague‐Dawley rats (Gill et al., 2015; Khurram et al., 2017). The *P*_di_ generated during airway occlusion in anesthetized Sprague‐Dawley rats of the same sex/age and weight range is ~30 cm H_2_O across studies (Fogarty et al., 2023; Gill et al., 2015; Khurram et al., 2017; Seven et al., 2018). On balance, it appears that *P*_di_ amplitude sighs are generally equivalent to those generated during airway occlusion.

The post‐sigh inter‐breath intervals were longer compared to inter‐breath intervals for eupnea both PRE‐ and POST‐C_2_SH. In rats, it appears that that the post‐sigh respiratory reset is almost immediate, compared to cats, where 5–6 post‐sigh breaths remain depressed compared to normal eupnea (Cherniack et al., 1981). An important limitation of the present study is that *P*_di_ measurements were performed while animals were anesthetized, which largely abolishes the post‐inspiratory phase of breathing. It remains to be seen in rats whether the duration of the post‐sigh respiratory reset is influenced by post‐inspiration DIAm activity.

In the present study, the amplitude of *P*_di_ during sighs was reduced POST‐C_2_SH. Previously in rats, we reported that C_2_SH reduced the *P*_di_ generated during eupnea, hypoxia/hypercapnia and occlusion (Fogarty et al., 2023). This remarkable effect of C_2_SH on ventilatory *P*_di_ is consistent with past reports of reduced tidal volume during eupnea and augmented breaths in unanesthetized rats (Fuller et al., 2006, 2008). Importantly, with C_2_SH, reduced *P*_di_ during ventilatory behaviors may not be entirely dependent on impaired DIAm function. Reduced stiffening of the thoracic cage by the chest wall musculature (intercostals) during inspiration (Beth Zimmer et al., 2015; Denton & McKinlay, 2009) may impair *P*_di_ due to increased thoracic compliance. In other studies evaluating the impact of C_2_SH on sighs, the amplitude of DIAm EMG (Bezdudnaya et al., 2018) and tidal volume was reduced (Dougherty et al., 2012; Fuller et al., 2008; Golder et al., 2005). It is important to note that different behaviors of the DIAm are affected by C_2_SH differently depending on the method of assessment. For example, the amplitude of DIAm EMG during airway occlusion has been reported to be unchanged (Martinez‐Galvez et al., 2016) or reduced (Fogarty et al., 2023; Warren et al., 2018) following C_2_SH, depending on the normalization procedure. In the present study, we have focused on the immediate effects of C_2_SH; however, there is a large body of literature chronicling spontaneous recovery of the injured side in the weeks following the lesion (Brown et al., 2022; Fogarty et al., 2023; Fuller et al., 2006, 2008; Goshgarian et al., 1991; Gransee et al., 2015, 2017; Hernandez‐Torres et al., 2017; Mantilla, Gransee, et al., 2013; Mantilla, Greising, et al., 2013; Martinez‐Galvez et al., 2016; Miyata et al., 1995; Moreno et al., 1992; Sieck et al., 2021; Singh et al., 2012). In rats, we have consistently observed ~30% spontaneous recovery in DIAm EMG during eupnea between 7‐ and 14‐days POST‐C_2_SH (Brown et al., 2022; Fogarty et al., 2023; Gransee et al., 2015; Hernandez‐Torres et al., 2017; Mantilla, Gransee, et al., 2013; Martinez‐Galvez et al., 2016; Sieck et al., 2021). Notably, the C_2_SH lesion in rat spares respiratory premotor inputs from the contralateral side and local interneurons (Fogarty & Sieck, 2020), which may be provide the substrate for de novo synaptogenesis and recovery (Gransee et al., 2017; Hadley et al., 1999; Mantilla et al., 2012; Porter, 1895; Streeter et al., 2017, 2020; Sunshine et al., 2020; Zhou & Goshgarian, 1999). Enhancement of this phenomena has been shown to occur by promoting neurotrophic, neuromodulatory, and synaptic formations, which can increase the level of eupneic recovery to >80% (Fogarty et al., 2023; Fuller et al., 2003; Gransee et al., 2013, 2015; Hernandez‐Torres et al., 2017; Mantilla, Gransee, et al., 2013; Martinez‐Galvez et al., 2016; Sieck et al., 2021; Wollman et al., 2020; Zhou & Goshgarian, 1999). However, few of these (Fogarty et al., 2023) have evaluated recovery across the full gamut of ventilatory and non‐ventilatory behaviors.

It is likely that spontaneous sighs utilize or interact with the canonical brainstem respiratory centers (Housley et al., 1970; Ramirez, 2014; Ramirez et al., 2022; Soltysik & Jelen, 2005). The results of the present study indicate that the generation of *P*_di_ during sighs is sensitive to C_2_SH removal of ipsilateral descending excitatory input to PhMNs. The *P*_di_ generated POST‐C_2_SH are in stark contrast to the *P*_di_ generated following unilateral phrenicotomy, where *P*_di_ generated during eupnea and hypoxia/hypercapnia are preserved with an increase in contralateral DIAm EMG, while the *P*_di_ generated during sigh and airway occlusion are reduced, with no compensatory increase in DIAm EMG on the uninjured side (Gill et al., 2015; Khurram et al., 2017). Following C_4_ unilateral contusion, the *P*_di_ generated during airway occlusion is reduced (Khurram et al., 2019), with no effects on DIAm EMG (Rana et al., 2017). Differences in the effects of C_2_SH and unilateral C_4_ contusion are likely related to the differential disruption of descending excitatory inputs to PhMN and the extent of contusion induced PhMN death, for example, with C_2_SH the extent of sparing of ipsilateral versus contralateral inputs to PhMNs is exceedingly important (Fogarty & Sieck, 2020; Rana et al., 2020; Tai & Goshgarian, 1996). With C_4_ contusion, frank PhMN death and DIAm denervation occurs on the injured side (Alvarez‐Argote et al., 2016; Rana et al., 2017) together with disruption of synaptic inputs to some PhMNs below the lesion.

Overall, this study shows that, in the absence of winnowing out non‐“stereotypical” sighs, the sigh is quite variable within and across rats and is highly sensitive to C_2_SH. Likewise the *P*_di_ of the post‐sigh breath is also affected by C_2_SH. In the spinal cord injury population, maintenance of cardiorespiratory health is essential (Fogarty & Sieck, 2020; Raab et al., 2021; Randelman et al., 2021), with sighs highly important in maintaining lung ventilatory function (Housley et al., 1970; Katagiri et al., 1998; Lieske et al., 2000; Ramirez et al., 2022; Riede et al., 2020; Wuyts et al., 2011). Our results of reduced sigh *P*_di_ amplitude likely impair the effectiveness of this behavior to stave of lung atelectasis. The positive effects of breathing exercises (e.g., breath‐stacking) post cervical spinal cord injury may be one way to accomplish what spontaneous sigh cannot in this population (Jeong & Yoo, 2015), although this probably is insufficient to mitigate any risks of pneumonias and airway infections due to impaired coughing (Berlowitz et al., 2016).

Our study also has important consequences for respiratory neurobiology in general, particularly with using sighs as an exemplar for the function of the entire respiratory neuromotor system. We caution against experimental approaches where atypical sighs are arbitrarily excluded and then subsequently used as a normalization, as this may artificially reduce variability, essentially doing nothing other than normalizing to twice eupnea. This is particularly important for conditions where sighs and the post‐sigh breaths are particularly vulnerable (e.g., Rett syndrome) (Fogarty, 2023; Huang et al., 2016; Robinson et al., 2012; Voituron et al., 2010). Here, we show that using an inclusive shape‐based, not amplitude‐based sigh sampling, we are able to capture the true variability within the behavior while also being sensitive enough to see an effect of injury. In the future as evaluations of sigh become less phenomenological, with more attention paid to their genesis within neural circuits, it will be important to pay attention to both the sigh itself and the immediate post‐sigh breath, as various species exhibit different *P*_di_ and temporal relationships to eupnea and maximal DIAm behaviors.

## FUNDING INFORMATION

This work was supported by National Institutes of Health grants HL146114 (GCS) and HL166204 (MJF).

## CONFLICT OF INTEREST STATEMENT

None of the authors has any conflicts of interest, real nor perceived, to disclose.

## ETHICS STATEMENT

All procedures were performed in accordance with the American Physiological Society's Animal Care Guidelines and the National Institutes of Health (NIH) guide for use and care of laboratory animals. These procedures were approved by the Institutional Animal Care and Use Committee (IACUC) at Mayo Clinic (A00003105‐17‐R20).
